# Effects of photobiomodulation on trismus in head and neck cancer patients after radiotherapy: a prospective, randomized, triple-blind, placebo-controlled clinical trial

**DOI:** 10.1007/s10103-026-04834-3

**Published:** 2026-03-14

**Authors:** Marcela Maria Fontes Borges Franco, André Alves Crispim, Gabriella Alves Julião Costa, Giulianna Barreto Aparecida, José Fernando Bastos Moura, Cássia Emanuella Nóbrega Malta, Mário Rogério Lima Mota, Paulo Goberlânio Barros Silva

**Affiliations:** 1 Federal University of Ceará, Fortaleza, Brazil; 2 Haroldo Juaçaba Hospital, Ceará Cancer Institute, Fortaleza, Brazil; 3 Unichristus, Fortaleza, Brazil

**Keywords:** Trismus, Radiotherapy, Head and neck cancer, Low-level light therapy, photobiomodulationd

## Abstract

To evaluate the effectiveness of photobiomodulation therapy (PBMT) in the prevention of trismus in patients undergoing Intensity-Modulated Radiotherapy (IMRT) and Volumetric Modulated Arc Therapy (VMAT), through a randomized, triple-blind, placebo-controlled clinical trial. A total of 46 patients undergoing radiotherapy (RT) for head and neck cancer (HNC) were included. Patients were equally and randomly allocated to PBMT and PBMT placebo groups and were evaluated daily by measuring mouth opening, pain during mouth opening, and pain on palpation of the masticatory muscles using the Visual Analog Scale (VAS). An extraoral infrared laser (~ 808 nm), power of 0.1 W, energy of 3 J, 30 s (107 J/cm²) per point was used, applied extraorally to the anterior temporalis (AT), masseter (MM), and temporomandibular joints (TMJ), and intraorally to the medial pterygoid muscle (MP). The Food Quality Inventory (ASGP) and quality of life analysis (QoL) (OHIP-14) were also applied. Statistical analysis included Student’s t-test, Mann-Whitney test, and linear regression models (*p* < 0.05). Patients in the PBMT group showed a significantly smaller reduction in mouth opening during radiotherapy compared to the placebo group (*p* = 0.005), as well as a lower prevalence of masticatory muscle pain and grade 1 trismus (*p* = 0.002). No significant differences were observed between the groups regarding the decayed, missing, and filled teeth index (DMFT), the Oral Health Impact Profile-14 (OHIP-14), or the Anderson Symptom and Overall Performance scores (*p* > 0.05). Mouth opening did not differ between the groups at the six-month follow-up. Photobiomodulation therapy was effective in preventing trismus and masticatory muscle pain during radiotherapy.

Clinical Trial Registration

Clinical trial number: RBR-67qxnt9 (registered on 16/04/2025).

**Supplementary Information:**

The online version contains supplementary material available at 10.1007/s10103-026-04834-3.

## Introduction

Trismus refers to a restriction in mouth opening, most commonly defined as a maximum interincisal opening of less than 35 mm [1]. In patients with head and neck cancer (HNC), trismus can affect up to 69% of patients after cancer treatment, and one of the main causal factors is radiation therapy (RT) [1, 2]. Although this mechanism remains unclear, several studies suggest that radiation-induced trismus is strongly associated with the formation of free radicals, tissue damage, hypoxia, and an increase in inflammatory cytokines and chemokines, causing muscle tissue damage and consequently inducing fibrosis of the masticatory muscles [3–5].

This often-underreported adverse effect can begin during or after radiotherapy (RT), typically becoming evident within the first six months and potentially persisting for up to ten years after completion of treatment [1, 6]. Several factors contribute to the risk of developing trismus in these patients, including advanced age, tumor location close to masticatory structures, high radiation doses (> 40 Gy), and prior surgical procedures or the use of concomitant chemotherapy and radiotherapy (CCP RT) [7–9]. Fibrosis resulting from radiation significantly reduces mandibular mobility, impairing oral functionality and overall quality of life [6, 10]. In more severe cases, trismus can lead to serious complications, such as malnutrition, aspiration pneumonia, and an increased risk of mortality [11, 12].

Photobiomodulation therapy has been explored as a potential treatment for trismus in patients receiving radiotherapy [13, 14]. The therapy is believed to promote anti-inflammatory and analgesic effects, thereby improving mouth opening [15, 16]. Preliminary studies indicate that PBMT may help reduce pain and inflammation in the masticatory muscles and temporomandibular joint (TMJ), potentially mitigating the effects of radiation [13, 14].

Although previous studies have demonstrated the therapeutic potential of PBMT for the treatment of trismus, evidence regarding its preventive role during RT remains limited. Therefore, this randomized, triple-blind, placebo-controlled clinical trial aimed to evaluate the efficacy of PBMT in preventing trismus in patients undergoing RT for head and neck cancer.

## Materials and methods

### Study design and ethical considerations

**Fig. 1:**
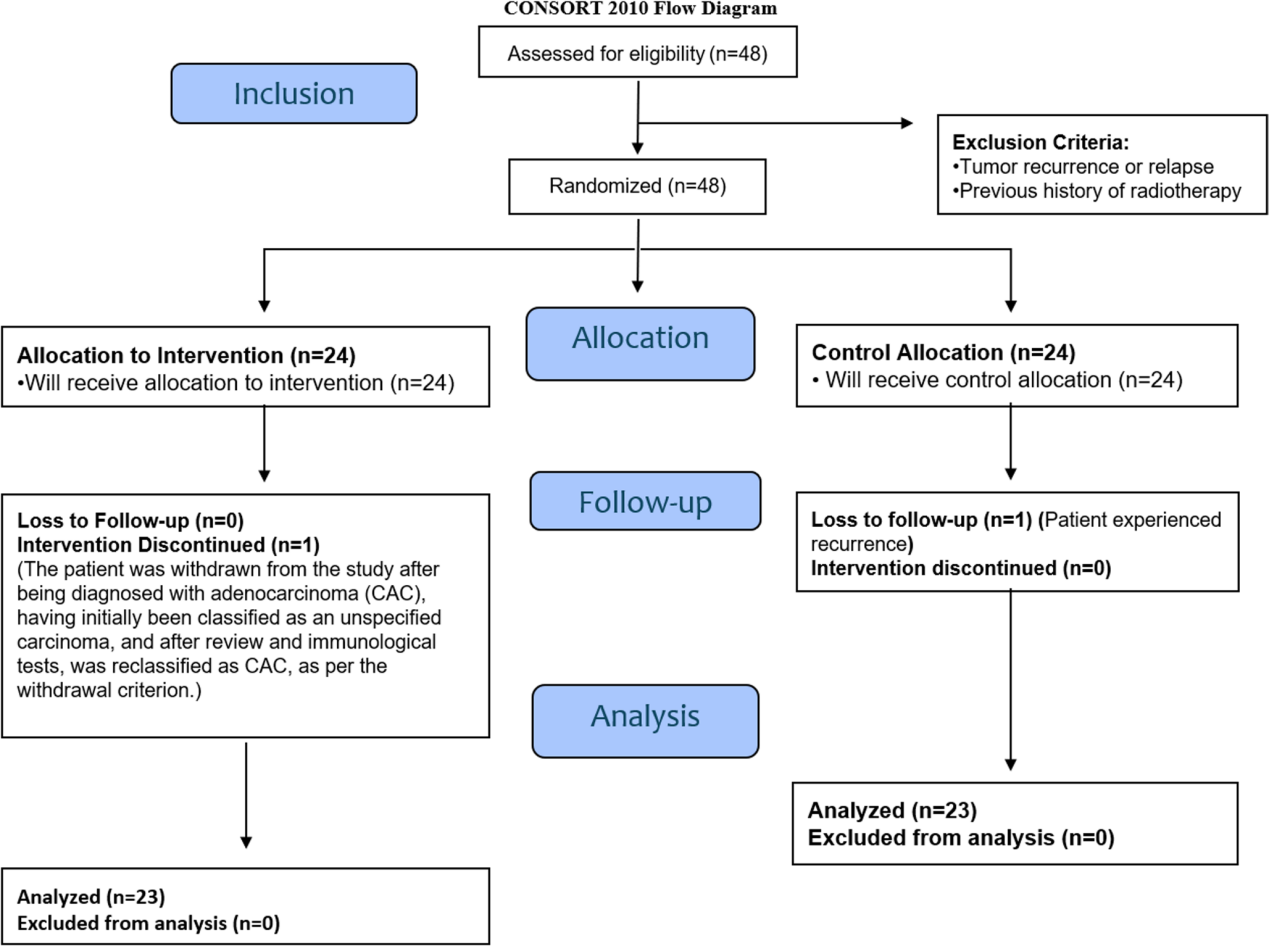
CONSORT Flowchart with inclusion, exclusion, and analysis criteria for patients with oral cancer treated with radiotherapy associated with chemotherapy and submitted to a randomized, triple-blind, placebo-controlled clinical trial. Legend: A total of 46 patients were evaluated, divided into two study groups (test and control). All patients received laser therapy; however, in the control group, the application was performed blindly using the method of turning the laser on and off

This is a phase II, randomized, triple-blind, placebo-controlled clinical trial registered in the Brazilian clinical trials registry (www.ensaiosclinicos.gov.br) (protocol RBR-67qxnt9) per the CONSORT guidelines for clinical trials (Fig. 1).

This study was approved by the Ethics Committee of the Haroldo Juaçaba Hospital (HHJ) Cancer Institute of Ceará (ICC) (protocol 5.432.685). All the ethical aspects expressed in Resolution 466 of 2012 of the National Health Council/Ministry of Health, which sets out the Guidelines and Regulatory Norms for research with human beings, and in accordance with the CONEP (National Research Ethics Commission) standard, were respected.

### Participants and clinical scenario: inclusion, exclusion, and withdrawal criteria

Patients over the age of 18 diagnosed with head and neck cancer in the nasopharynx, maxillae, oropharynx, hypopharynx, glottis, mouth, and salivary glands at stages I, II, III, or IV with a mouth opening equal to or greater than 35 mm who had been indicated for CCP RT were included. Radiotherapy could be shown as adjuvant, palliative, or curative and may or may not be associated with chemotherapy, immunotherapy, or biological therapies.

Patients with tumor recurrence, a previous history of radiotherapy in the head and neck region, patients with some involvement of the tumor in the masticatory muscles, and patients whose RT dose was < 40 Gy [14] were excluded. Patients who withdrew from treatment or the study required a change in the therapeutic protocol, developed extreme toxicity, or died were excluded from the study. All the patients were treated at the dental outpatient clinic in the radiotherapy department of the Haroldo Juaçaba Hospital, Ceará Cancer Institute.

### Study groups and experimental protocol

Clinical-pathological and sociodemographic data were collected after signing the Free and Informed Consent Form and agreeing to participate in the study. The Visual Analog Scale (VAS) were used to define pain scores on palpation of the masticatory muscles and for pain during mouth opening.

The patient-generated subjective global assessment (PGSGA) and the OHIP-14 quality of life questionnaire were administered at baseline before the principal investigator administered the first dose of laser or placebo. The principal investigator was the only one with access to the pre-sealed envelope identified with the patient’s study entry number and randomly assigned to one of the two study groups: the control group or the test group at the time of the participant’s entry into the study. Participants were blinded to group allocation. Before the start of the intervention, the principal investigator performed a visual examination of the oral cavity using a head-mounted photophore. Subsequently, photobiomodulation therapy was administered according to the study protocol. Both groups also received the PBM protocol, but in the control group, the laser therapy was performed in a simulated way by switching the equipment on and off immediately to blind the patient and the assessor [17].

A Therapy XT laser (DMC, São Carlos, SP, Brazil) with 100 mW of continuous wavelength light output of 660 ± 10 nm (red) and 820 ± 10 nm (infrared) was used. The device has a tip with an area of 0.28 mm² (or 0.0028 cm²), which, during the protocol applications, was kept in light contact with the treated area. Patients were treated daily from D0 before the first RT session until DF, the last day of RT. The photobiomodulation therapy protocol was conducted in accordance with the recommendations of the Multinational Association for Supportive Care in Cancer and the International Society of Oral Oncology (MASCC/ISOO) for supportive care in head and neck cancer patients. Laser parameters, including wavelength, power, energy density, and application time, were defined based on the guidelines of the World Association for Photobiomodulation Therapy (WALT) [18, 19].

**Fig. 2:**
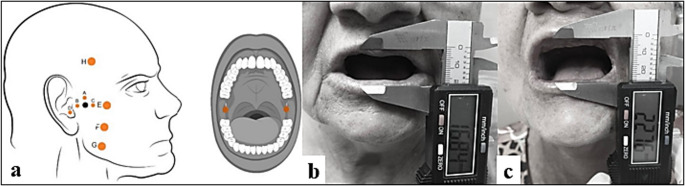
Illustration of the PBM protocol and clinical follow-up images. (**a**) Extraoral and intraoral anatomical points used for the application of low-level laser therapy according to the study by Borges et al. [14]. (**b**) Measurement of maximum mouth opening during RT. (**c**) Measurement of maximum mouth opening after RT

The photobiomodulation therapy protocol was conducted according to the predefined energy density and application points described by Borges et al. [14]. The infrared laser (~ 808 nm) was used extraorally, 0.1 W of power, 3 J of energy, the 30s (107 J/cm^2^) per point, totaling 270 s of application and 24 J of energy per side, the extraoral points were applied close to the region of the temporomandibular joint described as: (A) superior, (B) posterior and (C) anterior to the mandibular condyle, for the application of point (D) the patient was asked to open his mouth, and the laser was applied intra-auricularly towards the tragus. To target the masticatory muscles, the following points were applied: (E) upper, (F) middle, and (G) lower on the masseter muscle, a point (H) on the anterior portion of the temporal muscle. An intraoral point (I) was also applied to the medial pterygoid muscle using the infrared laser (~ 808 nm), 0.1 W of power, 3 J of energy, 30s (107 J/cm^2^) per point, totaling 30 s of application, and 3 J of energy per side. All applications (extraoral and intraoral) were carried out bilaterally, totaling 18 application points (Fig. 2 and Supplementary Material 1).

### Outcomes and data analysis

#### Evaluation of trismus and pain in the masticatory muscles

According to Costa et al. [16], the interincisal distance between the upper and lower central incisors was measured daily in millimeters using a caliper to assess maximum mouth opening and the presence of trismus. On the same days, the patient was also asked about their perception of pain in the masticatory muscles using the Visual Analog Scale (VAS), ranging from 0 to 10, where zero corresponds to no pain and 10 corresponds to maximum pain.

The *CTCAE v5.0* adverse event *criteria* scale was used to classify the degree of trismus clinically. Grade 1: Decreased range of motion of the masticatory muscles with adequate oral intake; Grade 2: Decreased range of motion of the masticatory muscles requiring small bites, soft foods, or purees; Grade 3: Decreased range of motion of the masticatory muscles with inability to eat adequately or hydrate orally. These assessments were also carried out every month up to 6 months after the end of RT treatment [14].

#### Collection of clinical-pathological and sociodemographic data

The Electronic Patient Record (EPP) was evaluated to collect clinical and pathological data, including age, tumor location, co-morbidities, drug use, psychological support, and nutritional guidance. Tumor characteristics, including pathological classification of tumor-nodule-metastasis (pTNM), histological type, and immunohistochemical analysis for p16, as well as sociodemographic data such as race, education level, occupation, geographic origin, place of birth, and family income, were collected.

Patients received daily treatment starting on day 0 (D0), corresponding to the day before the first radiotherapy session, until the final day of radiotherapy (DF). The patient was weighed on a conventional scale, and their weight was divided by their height squared to calculate their body mass index (BMI = mass / height²). These data were collected from the electronic medical records of the HHJ/ICC.

### Quality of life analysis

The OHIP-14 questionnaires are administered on day D0, at the start of the radiotherapy sessions, and on day DF, referring to the last radiotherapy session. The OHIP-14 is a subjective indicator that measures the disability, discomfort, and handicap attributed to the oral condition through self-assessment and its relationship with quality of life.

It consists of 14 questions and is a reduced version of the OHIP-49. It is also numbered on a Lickert-type scale, with answers ranging from (1) never, 2 (rarely), 3 (sometimes), 4 (repeatedly), and 5 (always) (Appendix II). Validated since the 1990 and widely used in dental research, it is divided into seven domains: (D1) Functional limitation (questions 1 and 2), (D2) Physical pain (questions 3 and 4), (D3) Psychological discomfort (questions 5 and 6), (D4) Physical limitation (questions 7 and 8), (D5) Psychological limitation (questions 9 and 10), (D6) Social limitation (questions 11 and 12) and (D7) Disability (questions 13 and 14). The seven domains comprise the overall quality of life scale (ranging from 14 to 70) [20].

### Food intake analysis

Food intake was analyzed at the beginning of the radiotherapy sessions (D0) and on the last day of radiotherapy (DF). The patients answered the Portuguese version of the patient-produced subjective global assessment (PGSGA). The ASGP is an inventory developed to assess the nutritional status of cancer patients, previously validated in Brazilian Portuguese. Composed of two blocks, one containing questions for the patient and the other containing assessments to be made by a health professional, the scale allows simple summations to obtain a nutritional status score for the patient being assessed. As it is a subjective scale, it was applied by the same professional to reduce observation bias [21].

### Sample calculation

Based on the study by BORGES et al. (2023) [14], they observed that the use of low-power laser photobiomodulation is capable of increasing daily mouth opening by + 1.69 ± 2.15 mm in patients with trismus related to head and neck radiotherapy, adopting 90% power and 95% confidence, it is estimated that it is necessary to evaluate 19 patients per study group (paired t-thesis). Given the possibility of sample loss, an additional 20% was added to this sample, giving 23 patients per study group.

#### Randomization and Blinding

Simple randomization was performed by an independent collaborator using the “=RAND()” function in Microsoft Excel (Microsoft Corporation^®^), allocating participants into two study groups (Groups A and B). After randomization, the randomization numbers were printed on sealed envelopes with the identification of which group they belonged to inside. They were opened only by the study’s principal investigator at the time of treatment.

The principal investigator needed the help of two collaborators blinded to the group to which the patient belonged for the clinical evaluation and collection of the questionnaires, thus making the study blind to the evaluators. In addition, the laser protocol was applied equally to both groups; however, the principal investigator simulated the application by turning the device on and off immediately, thus blinding the study to the patients. The evaluator and the supporting statistician were also unaware of the group to which each patient belonged. Therefore, only the principal investigator knew the groups to which the patients belonged, thus blinding the patient, the evaluators, and the statistician, making the study triple-blind.

### Statistical analysis

Categorical data were expressed as absolute and percentage frequencies and analyzed using Fisher’s exact or Pearson’s chi-square test. Quantitative data was expressed as mean and standard deviation, subjected to the Kolmogorov-Smirnov normality test, and compared using Student’s t-tests (parametric data) or Mann-Whitney tests (non-parametric data). Linear regression analysis was used to evaluate the variation in mouth opening and pain during mouth opening and on palpation of the masticatory muscles and TMJ during the radiotherapy cycles, and Cronbach’s alpha coefficient was used to analyze the internal consistency of the OHIP-14 quality of life questionnaire in each evaluation period. All the analyses were conducted using the Statistical Package for the Social Sciences (SPSS) software, version 20.0 for Windows, with a 95% confidence level.

## Results

### Clinical and radiotherapeutic characteristics of patients undergoing preventive PBM for trismus with low-level laser or placebo

Most participants were male (*n* = 19, 82.6%), and the mean age did not differ significantly between the groups (*p* = 0.421). The majority of patients self-identified as brown (PBMT: *n* = 17, 73.9%; placebo: *n* = 15, 65.2%; *p* = 0.522) and had incomplete primary education (PBMT: *n* = 15, 65.2%; placebo: *n* = 9, 39.1%; *p* = 0.133). Most participants reported a family income of up to one minimum wage (PBMT: *n* = 20, 87.0%; placebo: *n* = 21, 91.3%; *p* = 0.636) (Table 1).

**Table 1:** Sociodemographic and clinical-pathological profile of patients undergoing radiotherapy for head and neck cancers submitted to preventive photobiomodulation for trismus with low-power laser or placebo

	Group		*p*- Value
	PBMT	Placebo PBMT	
Sex			
Female	4 (17.4%)	4 (17.4%)	1.000
Male	19 (82.6%)	19 (82.6%)	
Age	60.57 ± 15.64	63.78 ± 10.80	0.421
Race			
White	6 (26.1%)	8 (34.8%)	0.522
Brown	17 (73.9%)	15 (65.2%)	
Education			
Not informed	2 (8.7%)	0 (0.0%)	0.133
Illiterate	2 (8.7%)	5 (21.7%)	
Primary education	15 (65.2%)	9 (39.1%)	
Secondary education	3 (13.0%)	8 (34.8%)	
Higher education	1 (4.3%)	1 (4.3%)	
Family income			
1sm	20 (87.0%)	21 (91.3%)	0.636
> 1sm	3 (13.0%)	2 (8.7%)	
Nutritional guidelines	22 (95.7%)	21 (91.3%)	0.550
Psychological support	19 (82.6%)	17 (73.9%)	0.475
Previous surgery	6 (26.1%)	4 (17.4%)	0.475
T			
T1	2 (8.7%)	2 (8.7%)	0.911
T2	6 (26.1%)	6 (26.1%)	
T3	7 (30.4%)	9 (39.1%)	
T4	8 (34.8%)	6 (26.1%)	
N			
N0	9 (39.1%)	14 (60.9%)	0.068
N1	1 (4.3%)	4 (17.4%)	
N2	11 (47.8%)	5 (21.7%)	
N3	2 (8.7%)	0 (0.0%)	
p16			
Negative	22 (95.7%)	20 (87.0%)	0.295
Positive	1 (4.3%)	3 (13.0%)	
Location			
Mouth	8 (34.8%)	7 (30.4%)	0.943
Oropharynx	7 (30.4%)	8 (34.8%)	
Laryngopharynx	4 (17.4%)	5 (21.7%)	
Nasopharynx	4 (17.4%)	3 (13.0%)	
Nasoenteral tube			
No	18 (78.3%)	20 (87.0%)	0.437
Yes	5 (21.7%)	3 (13.0%)	
RT dose			
< 69 Gy	8 (34.8%)	5 (21.7%)	0.459
69 Gy	7 (30.4%)	11 (47.8%)	
70 Gy	5 (21.7%)	5 (21.7%)	
Fractions			
Between 20 and 30	8 (34.8%)	5 (21.7%)	0.326
Between 31 and 35	15 (65.2%)	18 (78.3%)	
Type RT			
IMRT	11 (47.8%)	12 (52.2%)	0.768
VMAT	12 (52.2%)	11 (47.8%)	
RT interruptions	3 (13.0%)	5 (21.7%)	0.437
QT induction	2 (8.7%)	2 (8.7%)	1.000
RT duration (days)	47.70 ± 9.00	52.39 ± 8.91	***0.041***
CTCAE trismus score D1 to D35			
0	703 (87.3%)*	689 (85.6%)	***0.002***
1	56 (7.0%)	89 (11.1%)*	
2 or more	46 (5.7%)	27 (3.4%)	

*p < 0.05, Fisher’s exact test or Pearson’s chi-square (n, %) or Student’s t (mean ± SD). *RT* radiotherapy, *IMRT* intensity-modulated radiotherapy, *VMAT* volumetric arc radiotherapy, *D* day, *PBMT* photobiomodulation therapy. The number of CTCAE scores of trismus 0 in the PBMT group was 1.31 (95%CI = 1.06–1.62) times higher in the PBMT group (*p* = 0.002)

He most frequent clinical stages were T3 and T4 in both groups, with no significant difference between them (PBMT: T3 *n* = 7, 30.4%, T4 *n* = 8, 34.8%; placebo: T3 *n* = 9, 39.1%, T4 *n* = 6, 26.1%; *p* = 0.911). Regarding lymph node status, N0 disease was more frequent in the placebo group (*n* = 14, 60.9%) than in the PBMT group (*n* = 9, 39.1%), although this difference was not statistically significant (*p* = 0.068) (Table 1).

Most patients received radiotherapy doses ≥ 69 Gy, delivered in 31 to 35 fractions. No significant differences were observed between the groups in terms of total radiotherapy dose (*p* = 0.459), treatment technique (IMRT vs. VMAT; *p* = 0.768), treatment interruptions, or radiotherapy-related adverse events (*p* = 0.437) (Table 1).

The mean duration of radiotherapy was significantly shorter in the PBMT group (47.70 ± 9.00 days) than in the placebo group (52.39 ± 8.91 days; *p* = 0.041). Regarding trismus severity assessed by CTCAE between days 1 and 35, higher-grade scores (grades 1 and 2) were more frequently observed in the placebo group (11.1% and 3.4%, respectively) compared with the PBMT group (7.0% and 5.7%, respectively; *p* = 0.002). Additionally, the frequency of CTCAE grade 0 trismus was 1.31 times higher in the PBMT group (95% CI: 1.06–1.62) (Table 1).

### Photobiomodulation minimizes loss of mouth opening, pain, and trismus scores in irradiated head and neck patients

**Fig. 3:**
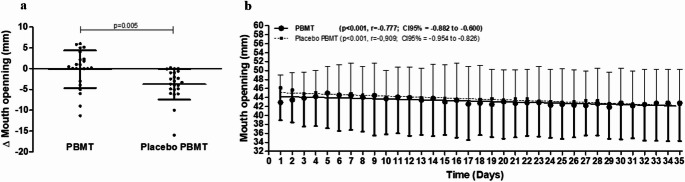
Influence of the preventive photobiomodulation protocol for trismus with low-level laser or placebo on the variation of mouth opening in patients undergoing radiotherapy for the treatment of head and neck cancers. (**a**) Variation of mouth opening (Δ Mouth Opening) in millimeters in the PBMT and Placebo PBMT groups after the treatment period. (**b**) Evolution of mouth opening (Mouth Opening) over time (days) in the PBMT and Placebo PBMT groups. *p < 0.05, Student’s t-test (mean ± SD) or linear regression model

The mean initial mouth opening of patients in the PBMT group was 42.89 ± 6.10 mm, while in the placebo group, it was 46.17 ± 7.28 mm (*p* = 0.104) on the first day (D1). There were no statistically significant differences in mouth opening between the PBMT and placebo PBMT groups on any of the days evaluated. However, both groups showed a slight reduction in mouth opening over the days, with a significant negative correlation with time that was stronger in the placebo PBMT group (*p* < 0.001, *r*= -0.909) than in the PBMT group (*p* < 0.001 *r*=-0.777) (Table 2; Fig. 3).

**Table 2:** Influence of preventive photobiomodulation protocol for trismus with low-power laser or placebo on pain variation during mouth opening in patients undergoing radiotherapy for head and neck cancer treatment

	Mouth opening		*p*-Value	Pain when opening the mouth		*p*- Value	Mouth opening vs. pain correlation	
	PBMT	Placebo PBMT		PBMT	Placebo PBMT		PBMT	Placebo PBMT
D1	42.89 ± 6.10	46.17 ± 7.28	0,104	0.00 ± 0.00	0.00 ± 0.00	1,000	*p* = 1.000 (*r* = 0.000)	*p* = 1.000 (*r* = 0.000)
D2	43.47 ± 6.01	45.66 ± 7.19	0,268	0.00 ± 0.00	0.00 ± 0.00	1,000	*p* = 1.000 (*r* = 0.000)	*p* = 1.000 (*r* = 0.000)
D3	43.86 ± 5.81	44.86 ± 7.35	0,611	0.00 ± 0.00	0.00 ± 0.00	1,000	*p* = 1.000 (*r* = 0.000)	*p* = 1.000 (*r* = 0.000)
D4	44.22 ± 5.82	45.04 ± 7.43	0,681	0.00 ± 0.00	0.00 ± 0.00	1,000	*p* = 1.000 (*r* = 0.000)	*p* = 1.000 (*r* = 0.000)
D5	44.98 ± 6.00	44.98 ± 7.87	1,000	0.00 ± 0.00	0.00 ± 0.00	1,000	*p* = 1.000 (*r* = 0.000)	*p* = 1.000 (*r* = 0.000)
D6	44.56 ± 6.72	44.56 ± 8.04	0,999	0.00 ± 0.00	0.00 ± 0.00	1,000	*p* = 1.000 (*r* = 0.000)	*p* = 1.000 (*r* = 0.000)
D7	44.44 ± 7.29	44.83 ± 8.08	0,864	0.00 ± 0.00	0.00 ± 0.00	1,000	*p* = 1.000 (*r* = 0.000)	*p* = 1.000 (*r* = 0.000)
D8	44.06 ± 6.83	44.52 ± 8.11	0,834	0.09 ± 0.42	0.13 ± 0.63	0,975	*p* = 0.463 (*r*=-0.161)	*p* = 0.098 (*r*=-0.354)
D9	44.44 ± 7.22	43.92 ± 8.40	0,823	0.09 ± 0.42	0.13 ± 0.63	0,975	*p* = 0.884 (*r* = 0.032)	*p* = 0.098 (*r*=-0.354)
D10	43.73 ± 6.33	43.65 ± 7.89	0,972	0.17 ± 0.58	0.26 ± 0.62	0,430	*p* = 0.114 (*r*=-0.338)	*p* = 0.336 (*r*=-0.210)
D11	44.10 ± 6.68	43.73 ± 7.74	0,860	0.30 ± 0.70	0.39 ± 0.72	0,542	*p = 0.004 (r=-0.576)	*p* = 0.524 (*r*=-0.140)
D12	43.99 ± 6.82	43.83 ± 8.38	0,943	0.48 ± 0.90	0.43 ± 0.73	0,890	*p* = 0.179 (*r*=-0.290)	*p* = 0.721 (*r*=-0.079)
D13	43.40 ± 7.14	43.94 ± 8.29	0,813	0.48 ± 0.90	0.43 ± 0.79	0,954	*p* = 0.240 (*r*=-0.255)	*p* = 0.991 (*r* = 0.003)
D14	43.82 ± 7.65	43.58 ± 8.20	0,920	0.48 ± 0.90	0.43 ± 0.79	0,954	*p* = 0.070 (*r*=-0.384)	*p* = 0.783 (*r*=-0.061)
D15	42.99 ± 8.30	43.79 ± 8.16	0,744	0.43 ± 0.90	0.39 ± 0.72	0,871	*p* = 0.151 (*r*=-0.309)	*p* = 0.422 (*r*=-0.176)
D16	43.43 ± 8.21	43.23 ± 8.20	0,935	0.35 ± 0.83	0.39 ± 0.72	0,573	*p = 0.009 (r=-0.535)	*p* = 0.457 (*r*=-0.163)
D17	42.55 ± 8.40	42.61 ± 8.13	0,981	0.43 ± 0.95	0.30 ± 0.63	0,891	*p = 0.007 (r=-0.546)	*p* = 0.755 (*r* = 0.069)
D18	42.74 ± 8.48	43.60 ± 8.02	0,727	0.39 ± 0.84	0.26 ± 0.69	0,474	*p = 0.002 r=-0.600)	*p* = 0.791 (*r*=-0.058)
D19	42.47 ± 7.82	43.76 ± 8.60	0,598	0.35 ± 0.78	0.26 ± 0.62	0,715	*p = 0.007 (r=-0.544)	*p* = 0.873 (*r* = 0.035)
D20	43.05 ± 7.11	43.49 ± 8.67	0,851	0.30 ± 0.76	0.35 ± 0.71	0,727	*p = 0.023 (r=-0.472)	*p* = 0.697 (*r*=-0.086)
D21	42.70 ± 7.46	43.64 ± 8.54	0,692	0.35 ± 0.78	0.30 ± 0.70	0,763	*p* = 0.068 (*r*=-0.387)	*p* = 0.903 (*r*=-0.027)
D22	42.84 ± 7.39	43.64 ± 8.19	0,732	0.39 ± 0.78	0.30 ± 0.70	0,552	*p = 0.031 (r=-0.451)	*p* = 0.860 (*r* = 0.039)
D23	42.86 ± 8.03	43.17 ± 7.92	0,894	0.52 ± 0.90	0.26 ± 0.62	0,283	*p* = 0.104 (*r*=-0.348)	*p* = 0.495 (*r* = 0.150)
D24	42.28 ± 7.95	43.11 ± 8.14	0,730	0.57 ± 0.90	0.26 ± 0.62	0,180	*p* = 0.157 (*r*=-0.305)	*p* = 0.677 (*r* = 0.092)
D25	42.44 ± 8.04	42.96 ± 8.19	0,827	0.65 ± 0.88	0.30 ± 0.63	0,121	*p* = 0.358 (*r*=-0.201)	*p* = 0.711 (*r* = 0.082)
D26	42.29 ± 7.92	43.00 ± 7.91	0,762	0.70 ± 0.88	0.35 ± 0.71	0,096	*p* = 0.355 (*r*=-0.202)	*p* = 0.732 (*r* = 0.076)
D27	42.18 ± 7.78	43.25 ± 7.72	0,643	0.70 ± 0.88	0.26 ± 0.62	***0***,***037***	*p* = 0.272 (*r*=-0.239)	*p* = 0.587 (*r*=-0.119)
D28	42.61 ± 7.94	43.29 ± 7.79	0,771	0.74 ± 0.92	0.26 ± 0.62	***0***,***033***	*p* = 0.177 (*r*=-0.292)	*p* = 0.583 (*r*=-0.121)
D29	41.85 ± 7.84	42.66 ± 7.87	0,730	0.70 ± 0.93	0.26 ± 0.62	0,058	*p* = 0.123 (*r*=-0.331)	*p* = 0.579 (*r*=-0.122)
D30	42.74 ± 7.81	42.47 ± 7.81	0,907	0.52 ± 0.90	0.35 ± 0.65	0,627	*p* = 0.082 (*r*=-0.370)	*p* = 0.854 (*r*=-0.040)
D31	42.16 ± 7.78	42.58 ± 7.68	0,857	0.57 ± 0.90	0.30 ± 0.63	0,298	*p* = 0.105 (*r*=-0.346)	*p* = 0.452 (*r*=-0.165)
D32	42.44 ± 7.82	42.35 ± 7.98	0,970	0.52 ± 0.95	0.39 ± 0.72	0,830	*p = 0.043 (r=-0.425)	*p* = 0.935 (*r*=-0.018)
D33	42.74 ± 7.59	42.54 ± 8.23	0,930	0.48 ± 0.90	0.43 ± 0.79	0,954	*p* = 0.081 (*r*=-0.371)	*p* = 0.930 (*r* = 0.019)
D34	42.74 ± 7.59	42.47 ± 8.22	0,906	0.48 ± 0.90	0.43 ± 0.79	0,954	*p* = 0.081 (*r*=-0.371)	*p* = 0.887 (*r* = 0.032)
D35	42.74 ± 7.59	42.43 ± 8.13	0,892	0.48 ± 0.90	0.43 ± 0.79	0,954	*p* = 0.081 (*r*=-0.371)	*p* = 0.887 (*r* = 0.032)
r	*-0*,*777*	*-0*,*909*		*0*,*846*	*0*,*698*			
95%CI	*-0.882 to -0.600*	*-0.954 to -0.826*		*0.714 to 0.919*	*0.475 to 0.836*			
*p*-Value	*0.001*	*0.001*		*0.001*	*0.001*			

*p < 0.05, linear regression model (time vs. outcome). *D* day, *PBMT* photobiomodulation therapy. The number of correlations between pain and mouth opening was 2.30 (95%CI = 1.73 to 3.05) times higher in the PBMT group (*p* = 0.005)

The mean change in mouth opening from the start to the end of RT was significantly more significant in the PBMT group (-0.15 ± 4.51 mm; *p* = 0.005) compared to the placebo group (-3.75 ± 3.69 mm). The difference between the two groups was 3.50 ± 1.21 mm. Only one patient in the PBMT group showed a reduction in mouth opening, while six patients in the placebo group had negative results in terms of mouth opening reduction (Fig. 3).

During the first seven days of follow-up (D1–D7), patients in both groups reported no pain (0.00 ± 0.00). From D8 onward, mean pain scores began to fluctuate but remained low throughout treatment. The PBMT group reported higher pain scores than the placebo group at only two time points (D7, *p* = 0.037; D28, *p* = 0.033).

In the PBMT group, increased mouth opening was significantly correlated with lower pain scores during mouth opening on specific days of radiotherapy, including D11 (*p* = 0.004, *r* = 0.576), D16 (*p* = 0.009, *r* = 0.535), D17 (*p* = 0.002, *r* = 0.600), D19 (*p* = 0.007, *r* = 0.544), D20 (*p* = 0.023, *r* = − 0.472), D22 (*p* = 0.031, *r* = 0.451), and D34 (*p* = 0.043, *r* = 0.425). No significant correlations were observed on any evaluation day in the placebo group (Table 2).

Trismus scores were predominantly grade 0 in both groups throughout the radiotherapy period, with no statistically significant differences between the PBMT and placebo PBMT groups at any evaluation time point (Supplementary Material 1).

### Photobiomodulation reduces pain on palpation of masticatory muscles in irradiated head and neck patients

PBMT resulted in a reduction of pain on specific days in the masseter muscles when compared to the placebo group. For the right masseter, PBMT reduced pain scores on D7 (*p* = 0.039), while for the left masseter, no significant differences were found. The average scores in the PBMT group remained consistently lower throughout the evaluation days compared to the placebo group (Table 3).

**Table 3:** Influence of a preventive photobiomodulation protocol for trismus with a low-power laser or placebo on the variation in pain on palpation of the masseter and temporal muscles in patients undergoing radiotherapy for head and neck cancer

	Masseter pain D		*p*- Value	Masseter pain E		*p*- Value	Temporal pain D		*p*- Value	Temporal pain E		*p*-
	PBMT	Placebo PBMT		PBMT	Placebo PBMT		PBMT	Placebo PBMT		PBMT	Placebo PBMT	Value
D1	0.13 ± 0.63	0.00 ± 0.00	0,317	0.26 ± 1.25	0.35 ± 1.67	0,975	0.00 ± 0.00	0.00 ± 0.00	1,000	0.00 ± 0.00	0.04 ± 0.21	0,317
D2	0.13 ± 0.63	0.00 ± 0.00	0,317	0.13 ± 0.63	0.35 ± 1.67	0,975	0.00 ± 0.00	0.00 ± 0.00	1,000	0.00 ± 0.00	0.09 ± 0.42	0,317
D3	0.00 ± 0.00	0.22 ± 0.74	0,153	0.26 ± 1.25	0.35 ± 1.67	0,975	0.04 ± 0.21	0.00 ± 0.00	0,317	0.00 ± 0.00	0.00 ± 0.00	1,000
D4	0.13 ± 0.63	0.22 ± 0.74	0,572	0.22 ± 1.04	0.30 ± 1.46	0,975	0.13 ± 0.46	0.00 ± 0.00	0,153	0.00 ± 0.00	0.43 ± 1.70	0,153
D5	0.13 ± 0.46	0.48 ± 1.41	0,569	0.22 ± 0.85	0.35 ± 1.67	0,590	0.04 ± 0.21	0.22 ± 1.04	0,975	0.00 ± 0.00	0.48 ± 1.34	***0***,***039***
D6	0.30 ± 0.88	0.61 ± 1.75	0,925	0.22 ± 0.85	0.48 ± 1.65	0,946	0.39 ± 1.67	0.43 ± 1.47	1,000	0.00 ± 0.00	0.48 ± 1.53	0,077
D7	0.00 ± 0.00	0.70 ± 1.77	***0***,***039***	0.26 ± 0.86	0.83 ± 2.61	0,925	0.30 ± 1.46	0.52 ± 1.50	0,334	0.00 ± 0.00	0.91 ± 2.21	***0***,***019***
D8	0.04 ± 0.21	0.70 ± 1.77	0,138	0.26 ± 0.86	0.65 ± 1.82	0,635	0.09 ± 0.42	0.26 ± 0.92	0,538	0.00 ± 0.00	0.52 ± 1.41	***0***,***039***
D9	0.17 ± 0.65	0.87 ± 1.87	0,182	0.22 ± 0.85	0.78 ± 1.88	0,218	0.00 ± 0.00	0.26 ± 0.92	0,153	0.00 ± 0.00	0.57 ± 1.53	0,077
D10	0.30 ± 0.82	0.52 ± 1.27	0,623	0.17 ± 0.58	0.65 ± 1.50	0,199	0.09 ± 0.42	0.26 ± 0.75	0,301	0.00 ± 0.00	0.57 ± 1.47	***0***,***039***
D11	0.48 ± 1.24	0.61 ± 1.50	0,947	0.26 ± 1.05	0.78 ± 2.30	0,368	0.13 ± 0.63	0.26 ± 0.75	0,323	0.13 ± 0.63	0.48 ± 1.31	0,291
D12	0.48 ± 1.24	0.48 ± 1.16	0,987	0.35 ± 1.47	0.70 ± 1.79	0,219	0.13 ± 0.63	0.13 ± 0.46	0,590	0.13 ± 0.63	0.65 ± 1.90	0,281
D13	0.57 ± 1.27	0.43 ± 1.08	0,715	0.43 ± 1.50	0.78 ± 1.95	0,425	0.13 ± 0.63	0.13 ± 0.46	0,590	0.13 ± 0.63	0.74 ± 1.91	0,155
D14	0.61 ± 1.56	0.52 ± 1.27	0,775	0.48 ± 1.70	0.70 ± 1.64	0,444	0.00 ± 0.00	0.13 ± 0.46	0,153	0.00 ± 0.00	0.57 ± 1.47	***0***,***039***
D15	0.43 ± 1.08	0.35 ± 0.83	0,947	0.39 ± 1.20	0.61 ± 1.73	0,474	0.00 ± 0.00	0.04 ± 0.21	0,317	0.00 ± 0.00	0.52 ± 1.34	***0***,***039***
D16	0.35 ± 1.03	0.30 ± 0.93	0,985	0.39 ± 1.20	0.52 ± 1.73	0,985	0.00 ± 0.00	0.48 ± 1.75	0,153	0.13 ± 0.63	0.74 ± 2.09	0,281
D17	0.43 ± 1.08	0.22 ± 0.60	0,623	0.39 ± 1.20	0.57 ± 1.73	0,699	0.00 ± 0.00	0.00 ± 0.00	1,000	0.13 ± 0.63	0.43 ± 1.47	0,521
D18	0.43 ± 1.08	0.30 ± 0.93	0,673	0.26 ± 0.62	0.52 ± 1.41	0,894	0.00 ± 0.00	0.00 ± 0.00	1,000	0.13 ± 0.63	0.43 ± 1.47	0,521
D19	0.30 ± 0.88	0.30 ± 0.93	1,000	0.35 ± 1.03	0.61 ± 1.78	0,686	0.00 ± 0.00	0.13 ± 0.63	0,317	0.00 ± 0.00	0.43 ± 1.47	0,153
D20	0.30 ± 0.88	0.35 ± 1.03	0,970	0.35 ± 1.03	0.74 ± 2.22	0,660	0.00 ± 0.00	0.26 ± 0.86	0,153	0.00 ± 0.00	0.65 ± 1.80	0,077
D21	0.30 ± 0.88	0.39 ± 1.20	0,970	0.26 ± 0.75	0.78 ± 2.26	0,610	0.00 ± 0.00	0.22 ± 0.74	0,153	0.00 ± 0.00	0.61 ± 1.75	0,077
D22	0.30 ± 0.88	0.35 ± 1.03	0,970	0.17 ± 0.65	0.78 ± 2.26	0,348	0.00 ± 0.00	0.13 ± 0.63	0,317	0.00 ± 0.00	0.61 ± 2.04	0,153
D23	0.39 ± 0.94	0.26 ± 0.75	0,660	0.30 ± 0.82	0.65 ± 1.97	0,673	0.00 ± 0.00	0.13 ± 0.63	0,317	0.00 ± 0.00	0.39 ± 1.37	0,153
D24	0.17 ± 0.65	0.35 ± 1.03	0,611	0.17 ± 0.65	0.65 ± 1.97	0,368	0.00 ± 0.00	0.13 ± 0.63	0,317	0.00 ± 0.00	0.52 ± 1.73	0,153
D25	0.26 ± 0.75	0.52 ± 1.27	0,610	0.26 ± 0.75	0.48 ± 1.38	0,673	0.00 ± 0.00	0.22 ± 0.74	0,153	0.00 ± 0.00	0.57 ± 1.88	0,153
D26	0.17 ± 0.65	0.39 ± 0.94	0,378	0.26 ± 0.75	0.65 ± 1.85	0,635	0.00 ± 0.00	0.13 ± 0.63	0,317	0.00 ± 0.00	0.70 ± 1.94	0,077
D27	0.26 ± 0.75	0.57 ± 1.47	0,623	0.26 ± 0.75	0.61 ± 1.56	0,610	0.00 ± 0.00	0.13 ± 0.63	0,317	0.00 ± 0.00	0.61 ± 1.78	0,077
D28	0.17 ± 0.65	0.57 ± 1.41	0,348	0.26 ± 0.75	0.61 ± 1.78	0,648	0.00 ± 0.00	0.22 ± 0.74	0,153	0.00 ± 0.00	0.65 ± 1.90	0,077
D29	0.26 ± 0.75	0.48 ± 1.24	0,648	0.17 ± 0.65	0.65 ± 1.85	0,358	0.00 ± 0.00	0.22 ± 0.74	0,153	0.00 ± 0.00	0.57 ± 1.56	0,077
D30	0.17 ± 0.65	0.70 ± 1.49	0,193	0.17 ± 0.65	0.65 ± 1.85	0,358	0.00 ± 0.00	0.26 ± 0.75	0,077	0.00 ± 0.00	0.70 ± 1.94	0,077
D31	0.22 ± 0.74	0.70 ± 1.49	0,199	0.22 ± 0.74	0.61 ± 1.78	0,388	0.00 ± 0.00	0.26 ± 0.75	0,077	0.00 ± 0.00	0.65 ± 1.77	***0***,***039***
D32	0.22 ± 0.74	0.74 ± 1.57	0,193	0.22 ± 0.74	0.78 ± 1.76	0,206	0.00 ± 0.00	0.26 ± 0.75	0,077	0.00 ± 0.00	0.65 ± 1.77	***0***,***039***
D33	0.22 ± 0.74	0.78 ± 1.68	0,193	0.22 ± 0.74	0.74 ± 1.68	0,212	0.00 ± 0.00	0.26 ± 0.75	0,077	0.00 ± 0.00	0.65 ± 1.77	***0***,***039***
D34	0.22 ± 0.74	0.78 ± 1.68	0,193	0.22 ± 0.74	0.74 ± 1.68	0,212	0.00 ± 0.00	0.26 ± 0.75	0,077	0.00 ± 0.00	0.65 ± 1.77	***0***,***039***
D35	0.22 ± 0.74	0.78 ± 1.68	0,193	0.22 ± 0.74	0.74 ± 1.68	0,212	0.00 ± 0.00	0.26 ± 0.75	0,077	0.00 ± 0.00	0.65 ± 1.77	***0***,***039***
r	0,095	***0***,***462***		-0,135	***0***,***493***		***-0***,***481***	0,189		-0,157	***0***,***521***	
95%CI	-0.245 to 0.415	***0.152 to 0.689***		-0.448 to 0.207	***0.192 to 0.710***		***-0.702 to -0.176***	-0.153 to 0.492		-0.466 to 0.185	***0.228 to 0.728***	
*p*-Value	0,584	***0***,***005***		0,439	***0***,***003***		***0***,***003***	0,274		0,365	***0***,***001***	

*p < 0.05, linear regression model (time vs. outcome). *D* day, *PBMT* photobiomodulation therapy

In the right masseter, both groups showed an increase in pain scores, but in the PBMT group this increase was 9.5% (*p* = 0.584) with values ​​ranging from 0.00 ± 0.00 to 0.22 ± 0.74, while in the placebo group 46.6% (*p* = 0.005) the scores ranged from 0.00 ± 0.00 to 0.78 ± 1.68. For the left masseter, there was a reduction of -13.5% (*p* = 0.439), ranging from 0.26 ± 1.25 to 0.22 ± 0.74; in the placebo group there was a significant increase in pain of 49.3% (*p* = 0.003) with scores ranging from 0.35 ± 1.67 to 0.74 ± 1.68 (Table 3).

There was no significant reduction in pain scores on specific days in the right and left temporal muscles. The mean daily reduction in pain scores on palpation of the right temporal muscle in the PBM group was marked − 48.1% (*p* = 0.003, ranging from 0.00 ± 0.00 to 0.00 ± 0.00. In the placebo group there was an increase in pain of 18.9% (*p* = 0.274). In the left temporal muscle there was also a reduction of -15.7% (*p* = 0.365) in pain scores on palpation, ranging from 0.00 ± 0.00 to 0.00 ± 0.0, while in the placebo group there was an increase in these scores of 52.1% (*p* = 0.001) throughout the RT treatment (Table 3).

In the medial pterygoid muscle on the right side, there was no significant reduction in pain scores on palpation on any evaluation day in the PBMT group (*p* > 0.05). However, in the medial pterygoid muscle on the left, there was a significant difference in pain scores on days D4 (*p* = 0.039), D5 (*p* = 0.019), D8 (*p* = 0.039), D9 (*p* = 0,039), D13 (*p* = 0.049), D30 (*p* = 0.043), D31 (*p* = 0.023), D32 (*p* = 0.023), D33 (*p* = 0.023), D34 (*p* = 0.023) and D35 (p-0.023) after PBM when compared to the placebo group (Table 4).

**Table 4:** Influence of preventive photobiomodulation protocol for trismus with low-power laser or placebo on the variation of pain on palpation in medial pterygoid muscles and temporomandibular joints in patients undergoing radiotherapy for head and neck cancers

	Medial pterygoid pain D		*p*- Value	Medial pterygoid pain E		*p*- Value	TMJ pain D		*p*- Value	TMJ pain AND		*p*-
	PBMT	Placebo PBMT		PBMT	Placebo PBMT		PBMT	Placebo PBMT		PBMT	Placebo PBMT	Value
D1	0.13 ± 0.46	0.35 ± 1.67	0,590	0.26 ± 1.25	0.35 ± 1.67	0,975	0.22 ± 1.04	0.00 ± 0.00	0,317	0.00 ± 0.00	0.00 ± 0.00	1,000
D2	0.13 ± 0.46	0.35 ± 1.67	0,590	0.00 ± 0.00	0.35 ± 1.67	0,317	0.22 ± 1.04	0.09 ± 0.42	0,975	0.00 ± 0.00	0.00 ± 0.00	1,000
D3	0.30 ± 1.06	0.61 ± 2.15	0,955	0.00 ± 0.00	0.57 ± 2.15	0,153	0.13 ± 0.63	0.13 ± 0.63	1,000	0.09 ± 0.42	0.30 ± 1.46	0,975
D4	0.48 ± 1.16	0.61 ± 2.15	0,495	0.00 ± 0.00	0.70 ± 2.16	***0***,***039***	0.13 ± 0.63	0.13 ± 0.63	1,000	0.13 ± 0.63	0.57 ± 2.02	0,521
D5	0.61 ± 1.75	0.83 ± 2.19	0,668	0.00 ± 0.00	0.70 ± 2.12	***0***,***019***	0.13 ± 0.63	0.00 ± 0.00	0,317	0.30 ± 1.02	0.30 ± 1.02	1,000
D6	0.74 ± 1.48	0.83 ± 2.23	0,747	0.22 ± 0.74	0.87 ± 2.30	0,225	0.22 ± 1.04	0.00 ± 0.00	0,317	0.17 ± 0.83	0.48 ± 1.56	0,312
D7	0.52 ± 1.24	0.83 ± 2.23	0,939	0.13 ± 0.63	1.00 ± 2.65	0,155	0.22 ± 1.04	0.04 ± 0.21	0,975	0.17 ± 0.83	0.74 ± 2.05	0,161
D8	0.57 ± 1.83	0.83 ± 2.23	0,454	0.00 ± 0.00	0.83 ± 2.04	***0***,***039***	0.00 ± 0.00	0.26 ± 0.75	0,077	0.09 ± 0.42	0.61 ± 1.78	0,155
D9	0.48 ± 1.70	1.00 ± 2.28	0,406	0.00 ± 0.00	1.00 ± 2.49	***0***,***039***	0.00 ± 0.00	0.35 ± 1.03	0,077	0.00 ± 0.00	0.52 ± 1.31	***0***,***039***
D10	0.52 ± 1.47	1.04 ± 2.46	0,434	0.09 ± 0.42	0.87 ± 2.03	0,075	0.00 ± 0.00	0.35 ± 1.03	0,077	0.00 ± 0.00	0.65 ± 1.82	***0***,***039***
D11	0.57 ± 1.62	0.96 ± 2.38	0,475	0.26 ± 1.25	1.04 ± 2.36	0,051	0.13 ± 0.63	0.22 ± 0.60	0,334	0.22 ± 1.04	0.78 ± 1.88	0,095
D12	0.48 ± 1.59	1.04 ± 2.14	0,157	0.26 ± 1.25	1.09 ± 2.41	0,051	0.13 ± 0.63	0.26 ± 0.75	0,323	0.22 ± 1.04	0.83 ± 2.21	0,095
D13	0.43 ± 1.44	1.09 ± 2.52	0,239	0.22 ± 1.04	1.04 ± 2.38	***0***,***049***	0.13 ± 0.63	0.30 ± 0.88	0,312	0.13 ± 0.63	0.83 ± 2.17	***0***,***049***
D14	0.35 ± 1.19	0.91 ± 1.76	0,134	0.30 ± 1.11	1.00 ± 2.45	0,218	0.09 ± 0.42	0.30 ± 0.88	0,291	0.22 ± 1.04	0.87 ± 1.91	0,051
D15	0.35 ± 1.19	0.70 ± 1.49	0,148	0.17 ± 0.58	0.83 ± 1.90	0,118	0.22 ± 1.04	0.13 ± 0.63	0,975	0.30 ± 1.46	0.48 ± 1.31	0,334
D16	0.17 ± 0.65	0.57 ± 1.27	0,206	0.26 ± 0.86	1.13 ± 2.67	0,126	0.22 ± 1.04	0.17 ± 0.83	0,975	0.13 ± 0.63	0.43 ± 1.24	0,301
D17	0.17 ± 0.65	0.52 ± 1.16	0,212	0.22 ± 0.74	0.87 ± 2.38	0,232	0.22 ± 1.04	0.17 ± 0.83	0,975	0.13 ± 0.63	0.48 ± 1.41	0,301
D18	0.35 ± 0.93	0.48 ± 1.16	0,660	0.35 ± 1.30	1.00 ± 2.47	0,225	0.22 ± 1.04	0.17 ± 0.83	0,975	0.09 ± 0.42	0.57 ± 1.78	0,281
D19	0.17 ± 0.65	0.52 ± 1.31	0,358	0.30 ± 1.02	0.96 ± 2.42	0,232	0.13 ± 0.63	0.13 ± 0.46	0,590	0.13 ± 0.63	0.70 ± 1.66	0,088
D20	0.35 ± 1.11	0.39 ± 1.20	0,985	0.13 ± 0.63	0.96 ± 2.42	0,084	0.13 ± 0.63	0.43 ± 1.31	0,291	0.13 ± 0.63	0.74 ± 1.84	0,088
D21	0.48 ± 1.34	0.43 ± 1.20	0,752	0.30 ± 1.02	0.91 ± 2.39	0,239	0.13 ± 0.63	0.35 ± 1.03	0,301	0.13 ± 0.63	0.78 ± 1.91	0,084
D22	0.17 ± 0.58	0.43 ± 1.20	0,388	0.22 ± 0.74	0.91 ± 2.61	0,378	0.13 ± 0.63	0.35 ± 1.03	0,301	0.13 ± 0.63	0.91 ± 2.15	0,081
D23	0.17 ± 0.58	0.48 ± 1.38	0,388	0.22 ± 0.74	0.70 ± 2.16	0,388	0.13 ± 0.63	0.35 ± 1.03	0,301	0.13 ± 0.63	0.65 ± 1.75	0,095
D24	0.17 ± 0.58	0.61 ± 1.47	0,212	0.22 ± 0.74	0.83 ± 2.39	0,378	0.13 ± 0.63	0.35 ± 1.03	0,301	0.13 ± 0.63	0.48 ± 1.34	0,173
D25	0.17 ± 0.58	0.57 ± 1.47	0,348	0.22 ± 0.74	0.83 ± 2.39	0,378	0.13 ± 0.63	0.26 ± 0.75	0,323	0.13 ± 0.63	0.70 ± 1.79	0,155
D26	0.17 ± 0.58	0.43 ± 1.08	0,368	0.13 ± 0.63	0.61 ± 1.75	0,291	0.13 ± 0.63	0.26 ± 0.75	0,323	0.13 ± 0.63	0.39 ± 0.94	0,173
D27	0.22 ± 0.74	0.83 ± 2.21	0,218	0.13 ± 0.63	0.70 ± 1.79	0,161	0.13 ± 0.63	0.30 ± 0.93	0,312	0.13 ± 0.63	0.52 ± 1.31	0,161
D28	0.09 ± 0.42	0.52 ± 1.41	0,155	0.13 ± 0.63	0.87 ± 2.40	0,161	0.13 ± 0.63	0.35 ± 1.03	0,301	0.13 ± 0.63	0.52 ± 1.41	0,167
D29	0.26 ± 0.69	0.52 ± 1.75	0,955	0.13 ± 0.63	0.83 ± 2.39	0,161	0.13 ± 0.63	0.39 ± 1.16	0,291	0.13 ± 0.63	0.52 ± 1.41	0,167
D30	0.30 ± 0.82	0.61 ± 1.67	0,881	0.13 ± 0.63	1.09 ± 2.52	***0***,***043***	0.13 ± 0.63	0.39 ± 1.16	0,291	0.13 ± 0.63	0.43 ± 1.16	0,173
D31	0.17 ± 0.58	0.65 ± 1.67	0,348	0.13 ± 0.63	1.13 ± 2.44	***0***,***023***	0.13 ± 0.63	0.35 ± 1.03	0,301	0.13 ± 0.63	0.48 ± 1.24	0,167
D32	0.09 ± 0.42	0.74 ± 1.98	0,149	0.13 ± 0.63	1.13 ± 2.44	***0***,***023***	0.13 ± 0.63	0.35 ± 1.03	0,301	0.13 ± 0.63	0.48 ± 1.24	0,167
D33	0.09 ± 0.42	0.78 ± 2.00	0,143	0.13 ± 0.63	1.13 ± 2.44	***0***,***023***	0.13 ± 0.63	0.35 ± 1.03	0,301	0.13 ± 0.63	0.52 ± 1.34	0,161
D34	0.09 ± 0.42	0.78 ± 2.00	0,143	0.13 ± 0.63	1.13 ± 2.44	***0***,***023***	0.13 ± 0.63	0.35 ± 1.03	0,301	0.13 ± 0.63	0.52 ± 1.34	0,161
D35	0.09 ± 0.42	0.78 ± 2.00	0,143	0.13 ± 0.63	1.13 ± 2.44	***0***,***023***	0.13 ± 0.63	0.35 ± 1.03	0,301	0.13 ± 0.63	0.52 ± 1.34	0,161
r	***-0***,***625***	-0,146		0,186	***0***,***513***		-0,094	***0***,***726***		0,098	0,192	
95%CI	***-0.793 to -0.368***	-0.457 to 0.196		-0.156 to 0.489	***0.217 to 0.723***		-0.414 to 0.246	***0.518 to 0.852***		-0.243 to 0.417	-0.150 to 0.494	
*p*-Value	***< 0.001***	0,402		0,282	***0***,***002***		0,589	***< 0.001***		0,574	0,267	

*p < 0.05, linear regression model (time vs. outcome). D = day; PBMT = photobiomodulation therapy

Over the 35 days of evaluation, there was a mean daily reduction of -62.5% (*p* < 0.001) in pain scores on palpation in the PBM group in the right medial pterygoid muscle, while on the left side, the mean daily reduction in the placebo group was − 14.6% (*p* = 0.402). In the left pterygoid muscle, there was an increase in pain scores in both groups, but in the PBMT group this increase was 18.6% (*p* = 0.282), while in the placebo group this increase was 51.3% (*p* = 0.002). However, on specific days, pain scores in the right and left medial pterygoid did not vary significantly on most days evaluated in both groups (*p* > 0.05), except on the specific days mentioned above (Table 4).

In the right TMJ, PBMT did not show statistically significant differences in pain scores on any of the days evaluated when compared to the placebo group (*p* > 0.05). However, on the left side, PBMT was significantly more effective in reducing pain on days D9 (*p* = 0.039), D10 (*p* = 0.039) and D13 (*p* = 0.049) compared to the placebo group. Linear regression indicated a mean daily reduction of -9.4% (*p* = 0.589) in the PBMT group, while in the placebo group there was a significant increase of 72.6% (*p* < 0.001). Pain scores in the left TMJ in the PBMT group increased in both groups, however in the BMTP group this increase was 9.8% (*p* = 0.574) and in the placebo group it was 19.2% (*p* = 0.267) over the 35 days of treatment (Table 4).

### Photobiomodulation to prevent trismus has little effect on oral health profile, nutritional intake, and quality of life

The DMFT index showed no statistically significant differences either within or between groups (*p* > 0.05). However, the ASGP increased significantly in both the PBMT group (*p* = 0.002) and the PBMT placebo group (*p* < 0.001), but there was no significant difference between the treatments (*p* > 0.05) (Table 5).

**Table 5:** Influence of preventive photobiomodulation protocol for trismus with low-power laser or placebo on oral health profile, nutritional intake, and quality of life in patients undergoing radiotherapy for head and neck cancer treatment

	Group		*p*- Value
	PBMT	Placebo PBMT	
CPO-D			
Initial	24.96 ± 9.35	26.52 ± 7.45	0,533
Final	25.04 ± 9.25	26.57 ± 7.37	0,540
*p*-Value	0,328	0,328	
ASGP			
Initial	5.83 ± 4.54	4.52 ± 4.61	0,339
Final	10.09 ± 4.90	11.04 ± 4.43	0,491
*p*-Value	***0***,***002***	***< 0***,***001***	
Weight (kg)			
Initial	61.98 ± 13.64	69.57 ± 14.87	0,078
Final	58.22 ± 12.29	64.78 ± 15.29	0,116
*p*-Value	***0***,***002***	***< 0***,***001***	
Weight variation (%)	94.19 ± 5.00	93.09 ± 6.86	0,537
OHIP-14			
Initial	18.00 ± 8.44	18.48 ± 7.01	0,532
Final	23.17 ± 12.67	23.91 ± 9.98	0,393
*p*-Value	***0***,***049***	***0***,***006***	
Functional limitation			
Initial	3.22 ± 2.17	2.96 ± 1.64	0,871
Final	4.61 ± 2.89	4.65 ± 2.19	0,683
*p*-Value	***0***,***022***	***0***,***003***	
Physical pain			
Initial	2.78 ± 1.48	2.87 ± 1.58	0,792
Final	4.22 ± 2.86	4.48 ± 2.39	0,500
*p*-Value	***0***,***008***	***0***,***002***	
Psychological discomfort			
Initial	2.30 ± 0.93	2.43 ± 1.31	0,699
Final	2.52 ± 1.34	2.96 ± 1.77	0,311
*p*-Value	0,197	0,167	
Physical disability			
Initial	2.87 ± 1.91	3.17 ± 2.25	0,831
Final	3.83 ± 3.07	3.87 ± 2.28	0,394
*p*-Value	0,180	0,244	
Psychological disability			
Initial	2.39 ± 0.99	2.57 ± 1.75	1,000
Final	2.48 ± 1.27	2.87 ± 1.52	0,206
*p*-Value	0,684	0,292	
Social disability			
Initial	2.26 ± 1.25	2.26 ± 0.75	0,334
Final	2.65 ± 1.40	2.83 ± 1.75	0,758
*p*-Value	0,340	0,058	
Handicap			
Initial	2.17 ± 0.83	2.22 ± 0.60	0,334
Final	2.87 ± 2.30	2.26 ± 0.75	0,866
*p*-Value	0,141	0,854	

*p < 0.05, Mann-Whitney test (analysis between groups) or Wilcoxon test (intra-group analysis). PBMT = photobiomodulation therapy

Regarding body weight, both groups showed a significant reduction over time (PBMT: *p* = 0.002; Placebo PBMT: *p* < 0.001), but there was no statistically significant difference in the percentage change in weight between the groups (*p* = 0.537) (Table 5).

As for the impact on quality of life, measured by the OHIP-14, there was a significant increase in the final scores in both groups (PBMT: *p* = 0.049; Placebo PBMT: *p* = 0.006), indicating a worsening in the perception of the oral impact. However, there was no statistically significant difference between the groups in the final values (*p* > 0.05) (Table 5).

The subscales of the OHIP-14 revealed that the dimensions of functional limitation and physical pain showed significant increases within both groups (*p* < 0.05), while the other dimensions, such as psychological discomfort, physical disability, psychological disability, social disability, and disadvantage, showed no significant changes within or between groups (*p* > 0.05) (Table 5).

The OHIP-14 scores showed small changes over time, with statistically significant differences in some questions. However, no robust differences were observed between the PBMT and Placebo PBMT groups (Supplementary Material 2).

### Influence of a preventive photobiomodulation protocol for trismus on mouth opening after the first six months of radiotherapy

No statistically significant differences were observed between the PBMT and PBMT placebo groups in any of the evaluated periods (M1 to M6; *p* > 0.05). Mouth opening showed a slight increase over time in both groups, with a positive correlation in the PBMT group (*r* = 0.720, 95% CI: -0.218 to 0.966; *p* = 0.106) and a negative, also non-significant, correlation in the PBMT placebo group (*r* = -0.726, 95% CI: -0.967 to 0.207; *p* = 0.101) (Table 6).

**Table 6:** Influence of preventive photobiomodulation protocol for trismus with low-power laser or placebo on the variation in mouth opening after the first six months of radiotherapy in patients with head and neck cancer tumors

	Mouth opening		*p*- Value	Pain when opening the mouth		*p*- Value
	PBMT	Placebo PBMT		PBMT	Placebo PBMT	
The period after RT (months)						
M1	40.22 ± 6.69	41.26 ± 6.33	0,589	0.57 ± 2.15	0.70 ± 1.99	0,655
M2	41.48 ± 6.63	40.48 ± 6.62	0,611	0.57 ± 2.15	0.91 ± 2.17	0,398
M3	41.65 ± 6.62	40.87 ± 7.65	0,713	0.83 ± 2.61	0.96 ± 2.33	0,712
M4	42.00 ± 7.38	40.91 ± 7.75	0,629	0.74 ± 2.38	0.91 ± 2.17	0,712
M5	41.48 ± 7.69	39.26 ± 7.58	0,330	0.48 ± 2.09	0.91 ± 2.17	0,388
M6	41.87 ± 7.30	40.00 ± 7.36	0,392	0.87 ± 2.38	0.91 ± 2.17	0,973
r	0,720	-0,726		0,383	0,580	
95%CI	-0.218 to 0.966	-0.967 to 0.207		-0.621 to 0.911	-0.4366 to 0.946	
*p*-Value	0,106	0,101		0,453	0,226	

*p < 0.05, linear regression model (time vs. outcome). *D* day, *PBMT* photobiomodulation therapy

Pain scores during mouth opening also showed no significant differences between the groups over the six-month follow-up (*p* > 0.05). Pain remained at low levels in both groups, with a weak positive correlation in the PBMT group (*r* = 0.383, 95% CI: -0.621 to 0.911; *p* = 0.453) and a moderate correlation in the PBMT placebo group (*r* = 0.580, 95% CI: -0.436 to 0.946; *p* = 0.226) (Table 6).

## Discussion

Trismus in patients with head and neck cancer is directly associated with a poorer quality of life and requires complex, multidisciplinary treatment [1, 6]. Previous studies have shown a positive effect of PBMT in reducing the severity of trismus in patients with head and neck cancer undergoing RT [13, 14]. This is the first clinical trial to investigate the use of low-level laser therapy as a preventive strategy for trismus related to radiotherapy in patients with head and neck cancer. The results demonstrated that the protocol was effective in mitigating the reduction in mouth opening and in reducing pain on palpation of the masticatory muscles when compared to the placebo group.

This condition is a serious public health problem, with the potential to evolve and cause severe consequences to the overall health status, ranging from malnutrition to increased mortality [11, 12]. Studies indicate that tumor extension in masticatory muscles, the presence of metastasis in cervical lymph nodes, previous surgeries, and radiotherapy contribute to the development of this condition [22–24]. Another important point is that trismus has a diagnosis and treatment, but once established, its reversal is complex and may persist for more than 10 years after the treatment of head and neck cancer [6]. Furthermore, there is a significant increase in the costs associated with the treatment of sequelae from antineoplastic therapy [25].

Although trismus is described as a late sequela of radiotherapy treatment, it is possible to observe that the loss of maximum mouth opening begins shortly after the onset of RT treatment [6]. In this study, we observed that both groups showed a reduction in mouth opening over the days, demonstrating a significant negative correlation with the RT duration. However, the variation in mouth opening over the RT sessions was significantly worse and more pronounced in the Placebo group.

The results obtained from the CTCAE trismus scores, between days D1 and D35, suggest that PBMT plays a relevant preventive role against the loss of mouth opening during radiotherapy. In the group treated with low-power laser, 87.3% of the patients maintained a score of 0, compared to 85.6% in the placebo group (*p* = 0.002). Furthermore, the PBMT group showed a lower frequency of progression to mild trismus (grade 1) and moderate/severe trismus (grade ≥ 2), with 7.0% and 5.7%, respectively, compared to 11.1% and 3.4% in the placebo group.

These findings indicate that PBMT not only contributed to maintaining mouth opening but also acted preventively in the progression of trismus. This result aligns with the average difference observed of 3.50 ± 1.21 mm more mouth opening in the PBMT group, reinforcing the protocol’s ability to control functional loss throughout the treatment.

A protocol for the treatment of radiotherapy-related trismus in head and neck cancer was developed and later adapted, demonstrating efficacy in a single-arm clinical trial for controlling this adverse effect [15, 16]. This protocol also proved to be effective in preventing trismus in the present study, attributing this result to the anti-inflammatory action of PBMT and suggesting that the local action of the laser may prevent the deleterious effects caused by RT during treatment and consequently minimize the loss of mouth opening that this population experiences after the initiation of treatment [16].

The treatment time was shorter in the PBMT group compared to the placebo group. Although this finding was not a primary outcome of the study, it is worth noting that treatment duration is considered a relevant prognostic parameter in patients with head and neck cancer (HNC) undergoing radiotherapy, as interruptions may be associated with higher recurrence rates, worse disease-free survival, and reduced overall survival [26]. Thus, although a causal relationship cannot be established, it is plausible to consider that PBMT may have indirectly contributed to the continuity of treatment.

Most studies report a gradual effect of radiation on the muscles, resulting in fibrosis and contracture, typically observed around nine weeks after the completion of treatment [27, 28]. However, in this study, we identified a reduction in maximum mouth opening early in the treatment, which persisted until its completion. These findings corroborate the study by Borges et al. [14], which also demonstrated the onset of this adverse effect during radiotherapy.

For the proper functionality of the rotational and translational movements of the mandible, neuromuscular balance is essential. When a muscle loses its range of motion, it begins to show signs of atrophy, even after just three days of impairment [29, 30]. We suggest that the daily inflammatory stimuli generated by radiotherapy progressively accumulate in the muscles and at the neural level, causing discomfort throughout the treatment and contributing to the development of progressive fibrosis in the future.

In the PBMT group, we observed a significant relationship between the improvement in mouth opening and the reduction in pain on specific days, suggesting that PBMT contributed to functional improvement associated with lower pain perception. On the other hand, in the Placebo PBMT group, no significant correlations were identified, indicating a less consistent response between mouth opening and pain. These findings reinforce the efficacy of PBMT, bringing analgesic, anti-inflammatory, and biostimulatory effects [13, 14].

In turn, the assessment of quality of life, food intake, and weight loss did not show a statistically significant difference between the groups, worsening similarly in both treatments. The discomfort of oncological treatment, the administration of concomitant therapies such as chemotherapy, and the complexity of the postoperative period for patients who underwent surgery appear to be more strongly associated with these events than the limitation of mouth opening [31].

In the assessments performed at six months of follow-up, no statistically significant differences were observed between the groups. However, a trend towards improvement was identified in the PBMT group, with a significant positive correlation between time and mouth opening (*r* = 0.720), suggesting a potential sustained functional effect of the intervention, even after the conclusion of the applications. It is important to consider that, at the end of RT, patients stop receiving photobiomodulation, which may diminish or terminate its anti-inflammatory and analgesic effects. These findings are particularly relevant given the literature evidence pointing to the difficulty in treating RT-related trismus in patients with head and neck cancer, as even functional therapies, such as physiotherapy, often fail to reverse this adverse effect [32].

Therefore, this study, despite its limitations, demonstrated that PBMT was effective in controlling the reduction in mouth opening and pain on palpation in patients undergoing radiotherapy, with minimal impact on quality of life, weight loss, and overall oral health. The main limitations include the relatively small sample size, the heterogeneity of tumor sites and oncologic treatments, and the limited post-radiotherapy follow-up period, which may restrict the assessment of long-term trismus progression. Additionally, the interruption of PBMT at the end of radiotherapy and the lack of control over functional factors, such as individual muscle conditioning, may have influenced.

## Conclusion

Preventive PBMT proved to be an effective strategy for minimizing trismus development in patients undergoing radiotherapy for head and neck cancer. The protocol helped preserve mouth opening and reduce pain, with minimal interference in quality of life, nutritional status, and overall oral health. These findings support the use of PBMT as a preventive adjunct during radiotherapy; however, further randomized clinical trials with larger sample sizes and longer follow-up periods are required to confirm its long-term benefits and establish standardized clinical protocols.

## Supplementary Information

Below is the link to the electronic supplementary material.

Supplementary Material 1

Supplementary Material 2

## Data Availability

No datasets were generated or analysed during the current study.

## Declarations

### Clinical trial registration

Clinical trial number: RBR-67qxnt9 (registered on 16/04/2025)
