# Supplementary material for: Effects of photobiomodulation on trismus in head and neck cancer patients after radiotherapy: a prospective, randomized, triple-blind, placebo-controlled clinical trial
Source: Lasers Med Sci. 2026 Mar 14;41(1):56. doi: 10.1007/s10103-026-04834-3 (PMC12987853; doi:10.1007/s10103-026-04834-3)
Supplement: Supplementary file 1 — Supplementary Material 1 [file 10103_2026_4834_MOESM1_ESM.docx]

| Parameter | Specification |
| --- | --- |
| Laser device | Therapy XT (DMC, São Carlos, SP, Brazil) |
| Emission mode | Continuous wave |
| Wavelength (red) | 660 ± 10 nm |
| Wavelength (infrared) | ~808–820 ± 10 nm |
| Output power | 100 mW (0.1 W) |
| Tip area | 0.28 mm² (0.0028 cm²) |
| Power density | 35.7 W/cm² |
| Energy per point | 3 J |
| Energy density (fluence) | 107 J/cm² |
| Application time per point | 30 seconds |
| Contact mode | Light contact with tissue |
| Application technique | Point-by-point |
| Extraoral application points | Temporomandibular joint (A–D); masseter muscle (E–G); temporalis muscle (H) |
| Intraoral application points | Medial pterygoid muscle (I) |
| Number of points per side | 9 |
| Total number of points | 18 (bilateral) |
| Total energy per side (extraoral) | 24 J |
| Total energy per side (intraoral) | 3 J |
| Total application time per session | 270 seconds |
| Treatment frequency | Daily |
| Treatment period | From day zero (D0, before first RT session) to the final day of radiotherapy (DF) |

**Supplementary material 1 (Table 1):** Laser irradiation parameters used in the photobiomodulation therapy protocol.

**Supplementary material 1 (table 2) :** Influence of preventive photobiomodulation protocol for trismus with low-power laser or placebo on CTCAE scores of mouth opening limitation in patients undergoing radiotherapy for treatment of head and neck cancers.

|  | **Group** | | **p-** |
| --- | --- | --- | --- |
|  | **PBMT** | **Placebo PBMT** | **Value** |
| **D1** |  |  |  |
| 0 | 23 (100.0%) | 23 (100.0%) | 1,000 |
| 1 | 0 (0.0%) | 0 (0.0%) |  |
| 2 | 0 (0.0%) | 0 (0.0%) |  |
| 3 | 0 (0.0%) | 0 (0.0%) |  |
| **D2** |  |  |  |
| 0 | 23 (100.0%) | 22 (95.7%) | 0,312 |
| 1 | 0 (0.0%) | 1 (4.3%) |  |
| 2 | 0 (0.0%) | 0 (0.0%) |  |
| 3 | 0 (0.0%) | 0 (0.0%) |  |
| **D3** |  |  |  |
| 0 | 23 (100.0%) | 21 (91.3%) | 0,148 |
| 1 | 0 (0.0%) | 2 (8.7%) |  |
| 2 | 0 (0.0%) | 0 (0.0%) |  |
| 3 | 0 (0.0%) | 0 (0.0%) |  |
| **D4** |  |  |  |
| 0 | 22 (95.7%) | 20 (87.0%) | 0,295 |
| 1 | 1 (4.3%) | 3 (13.0%) |  |
| 2 | 0 (0.0%) | 0 (0.0%) |  |
| 3 | 0 (0.0%) | 0 (0.0%) |  |
| **D5** |  |  |  |
| 0 | 22 (95.7%) | 20 (87.0%) | 0,295 |
| 1 | 1 (4.3%) | 3 (13.0%) |  |
| 2 | 0 (0.0%) | 0 (0.0%) |  |
| 3 | 0 (0.0%) | 0 (0.0%) |  |
| **D6** |  |  |  |
| 0 | 22 (95.7%) | 20 (87.0%) | 0,295 |
| 1 | 1 (4.3%) | 3 (13.0%) |  |
| 2 | 0 (0.0%) | 0 (0.0%) |  |
| 3 | 0 (0.0%) | 0 (0.0%) |  |
| **D7** |  |  |  |
| 0 | 21 (91.3%) | 20 (87.0%) | 0,636 |
| 1 | 2 (8.7%) | 3 (13.0%) |  |
| 2 | 0 (0.0%) | 0 (0.0%) |  |
| 3 | 0 (0.0%) | 0 (0.0%) |  |
| **D8** |  |  |  |
| 0 | 21 (91.3%) | 19 (82.6%) | 0,235 |
| 1 | 1 (4.3%) | 4 (17.4%) |  |
| 2 | 1 (4.3%) | 0 (0.0%) |  |
| 3 | 0 (0.0%) | 0 (0.0%) |  |
| **D9** |  |  |  |
| 0 | 21 (91.3%) | 18 (78.3%) | 0,362 |
| 1 | 1 (4.3%) | 4 (17.4%) |  |
| 2 | 1 (4.3%) | 1 (4.3%) |  |
| 3 | 0 (0.0%) | 0 (0.0%) |  |
| **D10** |  |  |  |
| 0 | 21 (91.3%) | 19 (82.6%) | 0,577 |
| 1 | 1 (4.3%) | 3 (13.0%) |  |
| 2 | 1 (4.3%) | 1 (4.3%) |  |
| 3 | 0 (0.0%) | 0 (0.0%) |  |
| **D11** |  |  |  |
| 0 | 21 (91.3%) | 19 (82.6%) | 0,577 |
| 1 | 1 (4.3%) | 3 (13.0%) |  |
| 2 | 1 (4.3%) | 1 (4.3%) |  |
| 3 | 0 (0.0%) | 0 (0.0%) |  |
| **D12** |  |  |  |
| 0 | 20 (87.0%) | 19 (82.6%) | 0,893 |
| 1 | 2 (8.7%) | 3 (13.0%) |  |
| 2 | 1 (4.3%) | 1 (4.3%) |  |
| 3 | 0 (0.0%) | 0 (0.0%) |  |
| **D13** |  |  |  |
| 0 | 20 (87.0%) | 19 (82.6%) | 0,893 |
| 1 | 2 (8.7%) | 3 (13.0%) |  |
| 2 | 1 (4.3%) | 1 (4.3%) |  |
| 3 | 0 (0.0%) | 0 (0.0%) |  |
| **D14** |  |  |  |
| 0 | 20 (87.0%) | 19 (82.6%) | 0,893 |
| 1 | 2 (8.7%) | 3 (13.0%) |  |
| 2 | 1 (4.3%) | 1 (4.3%) |  |
| 3 | 0 (0.0%) | 0 (0.0%) |  |
| **D15** |  |  |  |
| 0 | 20 (87.0%) | 19 (82.6%) | 0,527 |
| 1 | 2 (8.7%) | 3 (13.0%) |  |
| 2 | 0 (0.0%) | 1 (4.3%) |  |
| 3 | 1 (4.3%) | 0 (0.0%) |  |
| **D16** |  |  |  |
| 0 | 20 (87.0%) | 20 (87.0%) | 0,572 |
| 1 | 2 (8.7%) | 2 (8.7%) |  |
| 2 | 0 (0.0%) | 1 (4.3%) |  |
| 3 | 1 (4.3%) | 0 (0.0%) |  |
| **D17** |  |  |  |
| 0 | 19 (82.6%) | 20 (87.0%) | 0,527 |
| 1 | 3 (13.0%) | 2 (8.7%) |  |
| 2 | 0 (0.0%) | 1 (4.3%) |  |
| 3 | 1 (4.3%) | 0 (0.0%) |  |
| **D18** |  |  |  |
| 0 | 19 (82.6%) | 20 (87.0%) | 0,795 |
| 1 | 2 (8.7%) | 2 (8.7%) |  |
| 2 | 1 (4.3%) | 1 (4.3%) |  |
| 3 | 1 (4.3%) | 0 (0.0%) |  |
| **D19** |  |  |  |
| 0 | 19 (82.6%) | 20 (87.0%) | 0,795 |
| 1 | 2 (8.7%) | 2 (8.7%) |  |
| 2 | 1 (4.3%) | 1 (4.3%) |  |
| 3 | 1 (4.3%) | 0 (0.0%) |  |
| **D20** |  |  |  |
| 0 | 20 (87.0%) | 19 (82.6%) | 0,567 |
| 1 | 1 (4.3%) | 3 (13.0%) |  |
| 2 | 1 (4.3%) | 1 (4.3%) |  |
| 3 | 1 (4.3%) | 0 (0.0%) |  |
| **D21** |  |  |  |
| 0 | 20 (87.0%) | 19 (82.6%) | 0,567 |
| 1 | 1 (4.3%) | 3 (13.0%) |  |
| 2 | 1 (4.3%) | 1 (4.3%) |  |
| 3 | 1 (4.3%) | 0 (0.0%) |  |
| **D22** |  |  |  |
| 0 | 19 (82.6%) | 19 (82.6%) | 0,753 |
| 1 | 2 (8.7%) | 3 (13.0%) |  |
| 2 | 1 (4.3%) | 1 (4.3%) |  |
| 3 | 1 (4.3%) | 0 (0.0%) |  |
| **D23** |  |  |  |
| 0 | 19 (82.6%) | 19 (82.6%) | 0,753 |
| 1 | 2 (8.7%) | 3 (13.0%) |  |
| 2 | 1 (4.3%) | 1 (4.3%) |  |
| 3 | 1 (4.3%) | 0 (0.0%) |  |
| **D24** |  |  |  |
| 0 | 19 (82.6%) | 19 (82.6%) | 0,753 |
| 1 | 2 (8.7%) | 3 (13.0%) |  |
| 2 | 1 (4.3%) | 1 (4.3%) |  |
| 3 | 1 (4.3%) | 0 (0.0%) |  |
| **D25** |  |  |  |
| 0 | 19 (82.6%) | 19 (82.6%) | 0,753 |
| 1 | 2 (8.7%) | 3 (13.0%) |  |
| 2 | 1 (4.3%) | 1 (4.3%) |  |
| 3 | 1 (4.3%) | 0 (0.0%) |  |
| **D26** |  |  |  |
| 0 | 19 (82.6%) | 20 (87.0%) | 0,795 |
| 1 | 2 (8.7%) | 2 (8.7%) |  |
| 2 | 1 (4.3%) | 1 (4.3%) |  |
| 3 | 1 (4.3%) | 0 (0.0%) |  |
| **D27** |  |  |  |
| 0 | 19 (82.6%) | 20 (87.0%) | 0,795 |
| 1 | 2 (8.7%) | 2 (8.7%) |  |
| 2 | 1 (4.3%) | 1 (4.3%) |  |
| 3 | 1 (4.3%) | 0 (0.0%) |  |
| **D28** |  |  |  |
| 0 | 19 (82.6%) | 20 (87.0%) | 0,795 |
| 1 | 2 (8.7%) | 2 (8.7%) |  |
| 2 | 1 (4.3%) | 1 (4.3%) |  |
| 3 | 1 (4.3%) | 0 (0.0%) |  |
| **D29** |  |  |  |
| 0 | 19 (82.6%) | 20 (87.0%) | 0,795 |
| 1 | 2 (8.7%) | 2 (8.7%) |  |
| 2 | 1 (4.3%) | 1 (4.3%) |  |
| 3 | 1 (4.3%) | 0 (0.0%) |  |
| **D30** |  |  |  |
| 0 | 19 (82.6%) | 20 (87.0%) | 0,795 |
| 1 | 2 (8.7%) | 2 (8.7%) |  |
| 2 | 1 (4.3%) | 1 (4.3%) |  |
| 3 | 1 (4.3%) | 0 (0.0%) |  |
| **D31** |  |  |  |
| 0 | 19 (82.6%) | 20 (87.0%) | 0,795 |
| 1 | 2 (8.7%) | 2 (8.7%) |  |
| 2 | 1 (4.3%) | 1 (4.3%) |  |
| 3 | 1 (4.3%) | 0 (0.0%) |  |
| **D32** |  |  |  |
| 0 | 19 (82.6%) | 21 (91.3%) | 0,698 |
| 1 | 2 (8.7%) | 1 (4.3%) |  |
| 2 | 1 (4.3%) | 1 (4.3%) |  |
| 3 | 1 (4.3%) | 0 (0.0%) |  |
| **D33** |  |  |  |
| 0 | 19 (82.6%) | 19 (82.6%) | 0,753 |
| 1 | 2 (8.7%) | 3 (13.0%) |  |
| 2 | 1 (4.3%) | 1 (4.3%) |  |
| 3 | 1 (4.3%) | 0 (0.0%) |  |
| **D34** |  |  |  |
| 0 | 19 (82.6%) | 19 (82.6%) | 0,753 |
| 1 | 2 (8.7%) | 3 (13.0%) |  |
| 2 | 1 (4.3%) | 1 (4.3%) |  |
| 3 | 1 (4.3%) | 0 (0.0%) |  |
| **D35** |  |  |  |
| 0 | 19 (82.6%) | 19 (82.6%) | 0,753 |
| 1 | 2 (8.7%) | 3 (13.0%) |  |
| 2 | 1 (4.3%) | 1 (4.3%) |  |
| 3 | 1 (4.3%) | 0 (0.0%) |  |

*p<0.05, Fisher's exact test or Pearson's chi-square. D = day; PBMT = photobiomodulation therapy.
