# Supplementary material for: Effects of photobiomodulation on trismus in head and neck cancer patients after radiotherapy: a prospective, randomized, triple-blind, placebo-controlled clinical trial
Source: Lasers Med Sci. 2026 Mar 14;41(1):56. doi: 10.1007/s10103-026-04834-3 (PMC12987853; doi:10.1007/s10103-026-04834-3)
Supplement: Supplementary file 2 — Supplementary Material 2 [file 10103_2026_4834_MOESM2_ESM.docx]

**Supplementary material 2:** Item-by-item analysis of the OHIP-14 questionnaire in patients treated with preventive photobiomodulation for trismus with low-power laser or placebo during head and neck radiotherapy.

|  | **Initial** | | **p-** | **Final** | | **p-** | **Initial vs Final** | |
| --- | --- | --- | --- | --- | --- | --- | --- | --- |
|  | **PBMT** | **Placebo PBMT** | **Value** | **PBMT** | **Placebo PBMT** | **Value** | **PBMT** | **Placebo PBMT** |
| **OHIP-14** |  |  |  |  |  |  |  |  |
| Q1 | 1.39±1.16 | 1.57±1.20 | 0,485 | 2.22±1.70 | 1.83±1.37 | 0,541 | ***0,016*** | 0,236 |
| Q2 | 1.83±1.61 | 1.39±1.08 | 0,369 | 2.39±1.83 | 2.83±1.75 | 0,381 | 0,165 | ***0,004*** |
| Q3 | 1.09±0.29 | 1.35±0.83 | 0,329 | 1.78±1.48 | 2.00±1.35 | 0,263 | ***0,026*** | ***0,011*** |
| Q4 | 1.70±1.26 | 1.52±1.04 | 0,820 | 2.43±1.70 | 2.48±1.31 | 0,755 | ***0,031*** | ***0,008*** |
| Q5 | 1.17±0.49 | 1.17±0.65 | 0,669 | 1.30±0.82 | 1.35±0.83 | 0,725 | 0,257 | 0,285 |
| Q6 | 1.13±0.46 | 1.26±0.75 | 0,611 | 1.22±0.67 | 1.61±1.16 | 0,228 | 0,414 | 0,168 |
| Q7 | 1.43±1.04 | 1.48±1.12 | 0,812 | 1.91±1.68 | 1.96±1.22 | 0,360 | 0,179 | 0,170 |
| Q8 | 1.43±1.08 | 1.70±1.40 | 0,634 | 1.91±1.56 | 1.91±1.35 | 0,756 | 0,180 | 0,435 |
| Q9 | 1.22±0.60 | 1.39±1.03 | 0,660 | 1.26±0.92 | 1.74±1.21 | 0,078 | 1,000 | 0,113 |
| Q10 | 1.17±0.58 | 1.17±0.83 | 0,590 | 1.22±0.74 | 1.13±0.46 | 0,946 | 0,785 | 0,655 |
| Q11 | 1.09±0.42 | 1.17±0.65 | 0,555 | 1.00±0.00 | 1.35±0.83 | ***0,039*** | 0,317 | 0,194 |
| Q12 | 1.17±0.83 | 1.09±0.42 | 0,975 | 1.65±1.40 | 1.48±1.04 | 0,903 | 0,196 | 0,066 |
| Q13 | 1.09±0.42 | 1.22±0.60 | 0,312 | 1.52±1.38 | 1.26±0.75 | 0,866 | 0,131 | 0,854 |
| Q14 | 1.09±0.42 | 1.00±0.00 | 0,317 | 1.35±0.93 | 1.00±0.00 | 0,077 | 0,194 | 1,000 |
| **Cronbach's α** | **0,923** | **0,830** |  | **0,917** | **0,884** |  |  |  |

*p<0.05, Mann-Whitney test (between-group analysis) or Wilcoxon test (intra-group analysis). PBMT = photobiomodulation therapy; Q = OHIP-14 question.
